# Graphene/PVDF Composites for Ni-rich Oxide Cathodes toward High-Energy Density Li-ion Batteries

**DOI:** 10.3390/ma14092271

**Published:** 2021-04-27

**Authors:** Chang Won Park, Jung-Hun Lee, Jae Kwon Seo, Weerawat To A Ran, Dongmok Whang, Soo Min Hwang, Young-Jun Kim

**Affiliations:** 1 School of Advanced Materials Science and Engineering, Sungkyunkwan University, Suwon 16419, Korea; ason22@naver.com (C.W.P.); dwhang@skku.edu (D.W.); 2 AEB Cell Development Team, Samsung SDI Co., LTD, Yongin 17084, Korea; 3 SKKU Advanced Institute of Nano Technology (SAINT), Sungkyunkwan University, Suwon 16419, Korea; lee40003@naver.com (J.-H.L.); kwon4014@naver.com (J.K.S.); weerawat@skku.edu (W.T.A.R.); 4 Department of Nano Engineering, Sungkyunkwan University, Suwon 16419, Korea

**Keywords:** lithium-ion batteries, Ni-rich cathodes, graphene, PVDF, electrode density, scanning probe microscopy

## Abstract

Li-ion batteries (LIBs) employ porous, composite-type electrodes, where few weight percentages of carbonaceous conducting agents and polymeric binders are required to bestow electrodes with electrical conductivity and mechanical robustness. However, the use of such inactive materials has limited enhancements of battery performance in terms of energy density and safety. In this study, we introduced graphene/polyvinylidene fluoride (Gr/PVdF) composites in Ni-rich oxide cathodes for LIBs, replacing conventional conducting agents, carbon black (CB) nanoparticles. By using Gr/PVdF suspensions, we fabricated highly dense LiNi_0.85_Co_0.15_Al_0.05_O_2_ (NCA) cathodes having a uniform distribution of conductive Gr sheets without CB nanoparticles, which was confirmed by scanning spreading resistance microscopy mode using atomic force microscopy. At a high content of 99 wt.% NCA, good cycling stability was shown with significantly improved areal capacity (Q_areal_) and volumetric capacity (Q_vol_), relative to the CB/PVdF-containing NCA electrode with a commercial-level of electrode parameters. The NCA electrodes using 1 wt.% Gr/PVdF (0.9:0.1) delivered a high Q_areal_ of ~3.7 mAh cm^−2^ (~19% increment) and a high Q_vol_ of ~774 mAh cm^−3^ (~18% increment) at a current rate of 0.2 C, as compared to the conventional NCA electrode. Our results suggest a viable strategy for superseding conventional conducting agents (CB) and improving the electrochemical performance of Ni-rich cathodes for advanced LIBs.

## 1. Introduction

In an attempt to meet the global energy demand while maintaining sustainable development of society, rechargeable batteries have been considered to be effective solutions for renewable energy [1,2]. Lithium-ion batteries (LIBs) have been used as major power sources for a variety of applications, including portable electronics and electric vehicles, since having been devised in the 1990s [3,4,5,6,7]. Lower energy cost LIBs are desirable (i.e., <$125 kWh^−1^ for pack cost for electric vehicles) for providing a longer lifespan at an affordable cost [1,8]. Accordingly, most research has focused on the development of electrode materials and the electrode engineering via tuning the electrode parameters toward cycling-stable LIBs with high-energy density [4,5,6,7,8,9].

LIB electrodes are composed of a porous, composite-type film coated on a current collector foil, where the film contains active materials, carbonaceous conducting agents, and polymeric binders. Due to their low electrical conductivities, typical cathodes for LIBs require few percentages of nanoscale conductive additives, such as carbon blacks (CBs). However, employing CB nanoparticles in electrodes causes intrinsic challenges in electrode manufacturing processes, such as slurry formulation and coating/drying steps, due to their agglomeration behavior [10,11]. In addition, the low tap density of CB restricts an improvement of volumetric capacity (energy density) of electrodes. In practice, the slurry preparation step is decisive in a subsequent slurry coating process and the resulting electrochemical performance of electrodes [10]. Many studies on slurry processing have been conducted to improve rheological behavior and resulting electrode performance by modifying the methods and sequences of constituent mixing [10,12,13,14]. Nevertheless, the uniform dispersion of CB nanoparticles within a slurry and hence, their effective distribution in electrodes for ensuring the conductive pathways still remain challenging, even with smaller CB contents.

In this study, we used Gr nanosheets as a conducting agent in a Ni-rich oxide cathode, LiNi_0.85_Co_0.15_Al_0.05_O_2_ (NCA), replacing conventional CB nanoparticles for LIBs. By simply mixing an electrochemically exfoliated Gr dispersion and a polyvinylidene fluoride (PVdF) binder solution, we fabricated uniform Gr/PVdF suspensions with controlled Gr contents; this enabled uniform distribution of the Gr nanosheets within NCA electrodes due to the intimate interactions of the functional groups of Gr and the electronegative fluorine in PVdF. Surface probe microscopy on a cross section of the electrodes was conducted to differentiate the microstructure and intraparticle resistances. Using 1 wt.% Gr/PVdF suspension (Gr:PVdF = 0.9:0.1 in wt.%) without a conventional CB, we achieved highly dense NCA cathodes (99 wt.% NCA; electrode density (*ρ*)~3.8 g cm^−3^) that showed good cycling stability and higher areal capacity (Q_areal_) and volumetric capacity (Q_vol_) than those of a counterpart with a commercial-level of electrode parameters (96 wt.% NCA; *ρ*~3.3 g cm^−3^) at a current rate of 0.2 C.

## 2. Materials and Methods

### 2.1. Preparation of Gr/PVdF Suspensions

Gr nanosheets were prepared using an electrochemical exfoliation method. As the electrolyte, an aqueous solution containing 0.5 M ammonium sulfate ((NH_4_)_2_S_2_O_8_) was used, where two graphite foil electrodes were immersed in parallel at a fixed distance of 10 mm. When applying a DC voltage (+10 V) between the two graphite electrodes for about 10 min, a positively charged electrode was gradually exfoliated into Gr nanosheets, which was then collected through vacuum filtration, followed by washing with deionized water and ethanol. Gr/PVdF suspensions with various Gr contents (10–90 wt.%) were prepared in the following two steps. First, the Gr dispersion and PVdF (Solef^®^ 6020, Solvay, Brussels, Belgium) solution were prepared at concentrations of 2 wt.% and 10 wt.% in N-methyl-2-pyrrolidone (NMP), respectively. The two separate solutions were then prepared with a vortex mixer to form Gr/PVdF suspensions at different Gr contents (10–90 wt.%).

### 2.2. Electrochemical Testing

The cathode slurries were prepared by mixing 99 wt.% NCA powder (Samsung SDI, Yongin, Korea) and 1 wt.% Gr/PVdF suspensions, where the Gr content was varied from 0.1 to 0.9 wt.%. The as-prepared slurries were cast on a 20 μm-thick Al foil using a doctor blade method and dried in a convection oven at 80 °C for 2 h, followed by vacuum drying at 120 °C overnight. For comparison, NCA electrodes with commercial-level electrode parameters of NCA:CB (Super P^®^, Imerys Graphite & Carbon, Bironico, Switzerland):PVdF = 96:2:2 were prepared. The *ρ* was set as 3.8 (±0.1) g cm^−3^ and 3.3 (±0.1) g cm^−3^ for Gr-containing electrodes and CB-containing electrodes, respectively. The mass loading was set as ~16 mg cm^−2^ and it was increased to ~19 mg cm^−2^, to fabricate thicker electrodes. The electrodes were punched into discs with a diameter of 12 mm. Coin-type 2032 cells were assembled for electrochemical testing in an Ar filled glove box, with a lithium metal counter-electrode (16Φ) and polypropylene separator (Celgard 2400, Celgard, Charlotte, NC, USA). LiPF_6_ (1 M) in a solvent mixture of ethylene carbonate, ethyl methyl carbonate, and dimethyl carbonate at a volume ratio of 2:4:4 with 1.5% vinylene carbonate additive was used as the electrolyte. 

Electrochemical properties were measured using a WBCS3000L battery cycler (WonATech, Seoul, Korea). The formation process was conducted at 0.2 C (1C = 200 mAh g^−1^) in the voltage range of 2.75–4.3 V vs. Li^+^/Li for two cycles with a CC-CV charge mode using a 0.05 C cut-off. The discharge rate capability was measured at various current rates of 0.2–10 C after charging at 0.2 C with a CC-CV protocol using a 0.05 C cut-off. The cycle performance was evaluated at 30 and 60 °C in the voltage range of 2.75–4.3 V or 2.75–4.5 V vs. Li^+^/Li at a current rate of 0.5 C with a CC-CV charging protocol using a 0.05 C cut-off after two cycles of the formation process. Electrochemical impedance spectroscopy (EIS) was carried out using a biologic VSP-100 instrument in the frequency range of 1 MHz to 10 mHz with a voltage perturbation of 10 mV. The internal pressure change was monitored using a home-made gas cell system containing a cathode charged to 4.5 V vs. Li^+^/Li and 10 mL of a fresh electrolyte at 60 °C for 24 h in an Ar atmosphere.

### 2.3. Characterizations

The microstructure and Gr distribution were observed using field-emission scanning electron microscopy (SEM, JSM-7600F, JEOL Ltd., Tokyo, Japan). The samples were cross-sectioned using a cross-section polisher (SM-09010, JEOL Ltd., Tokyo, Japan). The in-plane resistance of NCA electrodes was measured using an electrode resistance measurement system (RM2610, Hioki, Nagano, Japan). The electrical resistance distribution inside the cross-sectioned electrodes was measured in scanning spreading resistance microscopy (SSRM) mode using atomic force microscopy (AFM, NX-10, Park systems, Suwon, Korea) equipped with a diamond-coated probe (CDT-NCHR, NANOSENSORS™, Neuchatel, Switzerland) with a nominal force constant of 80 N m^−1^ over a scan area of 10 × 10 µm^2^. By applying appropriate bias voltages (~1.5 V), the current passing through samples (between the tip and sample base, Al current collector) was recorded using a logarithmic current amplifier in SSRM mode.

## 3. Results and Discussion

**Figure 1:**
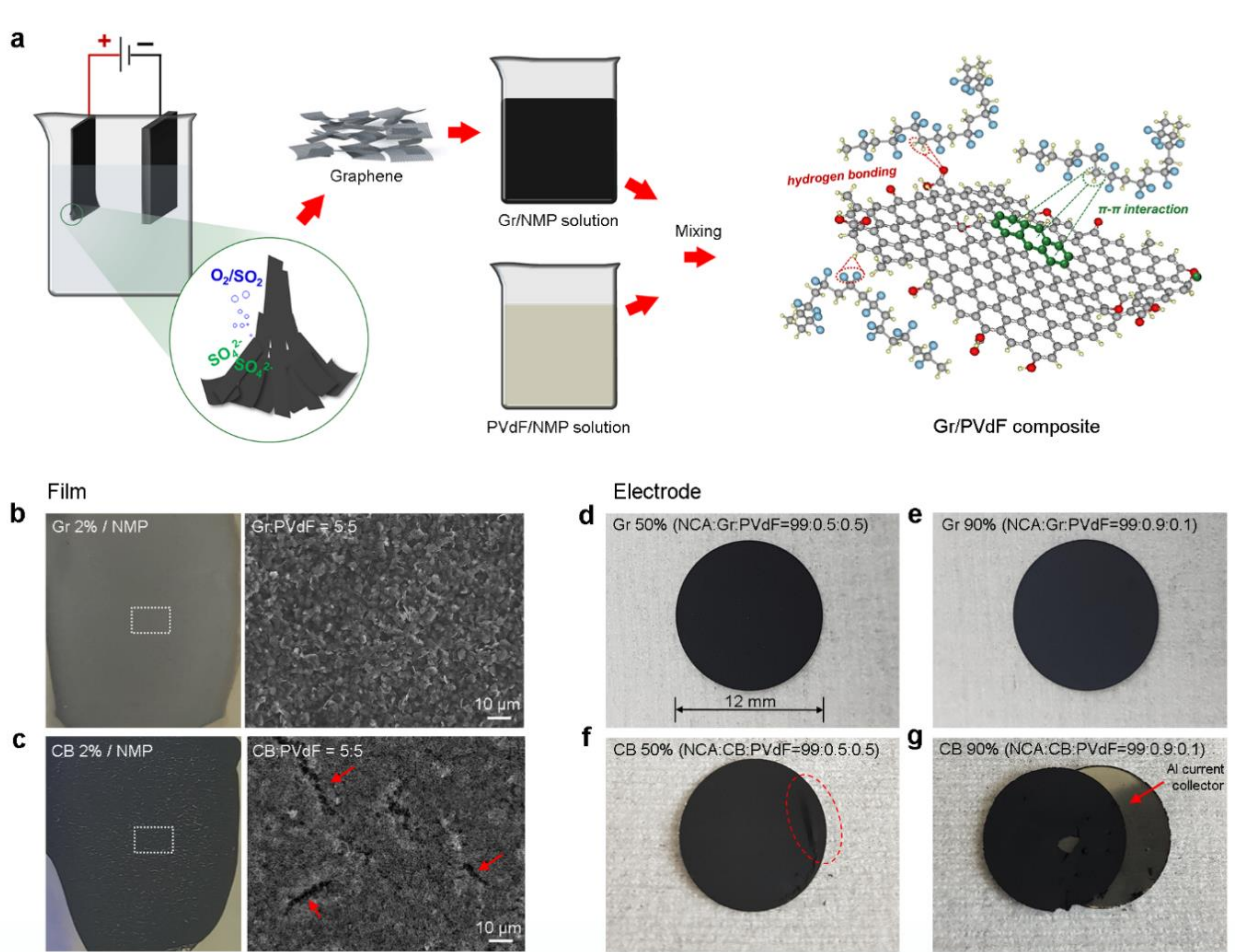
(**a**) Schematic of the preparation steps of Gr/PVdF suspensions and the bonding configurations between Gr and PVdF, where red, grey, ivory, and blue spheres indicate oxygen, carbon, hydrogen, and fluorine, respectively; digital images and SEM images of (**b**) Gr/PVdF and (**c**) CB/PVdF films, where the red arrows indicate microcracks on the film surface; and digital images of NCA electrodes with (**d**) 0.5 wt.% and (**e**) 0.9 wt.% Gr and (**f**) 0.5 wt.% and (**g**) 0.9 wt.% CB. The red dotted circle and red arrow indicate delamination from the Al current collector.

We prepared Gr dispersions by an electrochemical exfoliation method with commercially available graphite foils [15,16,17]. By applying a DC voltage (+10 V), a graphite foil was exfoliated into Gr sheets in an aqueous ammonium persulfate solution (0.5 M), due to the evolution of gases, such as SO_2_ and O_2_. The as-exfoliated Gr sheets were then collected by vacuum filtration and washing processes, followed by dispersion in a NMP solvent, as schematically depicted in Figure 1a. The exfoliated Gr sheets have lateral sizes of 0.1–2 μm and an average thickness of 4.6 nm (refer to Figure S1). The Gr surface/edges appeared to be partially oxygen-functionalized (C–OH/C–O/C=O) due to the oxidation of graphite by OH^–^ ions during the electrochemical process on the basis of XPS C 1s spectra (Figure S2) [16,17]. We performed confocal Raman mapping on the exfoliated Gr sheets, which showed the high-intensity G peak and 2D peak at ~1580 and ~2700 cm^−1^, respectively, with an intensity ratio of D to G (I_D_/I_G_) of ~0.3 (Figure S3), which is much lower than that of chemically or thermally reduced graphene oxides (I_D_/I_G_ = 1.2–1.5) [15,18,19].

Gr/PVdF suspensions were fabricated by simply mixing the Gr dispersion (2 wt.% Gr) and PVdF solution (10 wt.% in NMP) at different Gr contents (10–90 wt.%), speculating that two types of interactions between the Gr sheets and PVdF occur:―(i) hydrogen bonding between the oxygen functional groups (–O-C/–OH) on Gr and –CF_2_/–CH_2_ dipoles in PVdF and (ii) π–π interactions between the delocalized π electrons in Gr and –CH_2_ dipoles in PVdF―(see the right side of Figure 1a) [20,21,22,23,24,25]. A Gr/PVdF suspension (50:50 in wt.%) was cast on an Al foil and the film microstructure was compared to that of the film coated with a CB/PVdF suspension (50:50 in wt.%). As shown in Figure 1b,c, the Gr/PVdF film appeared to be highly homogeneous without noticeable protrusions and the plan-view SEM image showed uniform microstructure (Figure 1b). On the other hand, local protrusions and microcracks were found on the surface of the CB/PVdF film, as indicated by red arrows in Figure 1c. This difference in the microstructure between the two different type films could stem from the specific interactions between the Gr sheets and PVdF and the intrinsic agglomeration of CB in NMP (refer to Figure S4). The agglomeration of CB can cause intrinsic issues in slurry formation and coating/drying processes for electrode fabrication.

Typically, inactive components, such as CB and PVdF binder, have been used to build Ni-rich oxide cathodes; however, they should be minimized to enhance the energy density (volumetric capacity) of electrodes [26,27]. We fabricated electrodes having a high NCA content (99 wt.%) using Gr/PVdF and CB/PVdF suspensions (Figure 1d–g). The NCA electrodes composed of Gr/PVdF were robust without noticeable delamination from the Al foil current collector, even for the electrode containing a low PVdF content (0.1 wt.%; Figure 1e). By contrast, the NCA electrodes using the CB/PVdF were easily detached after electrode punching: the electrode containing 0.1 wt.% PVdF binder was completely delaminated from the current collector (Figure 1g). 

**Figure 2:**
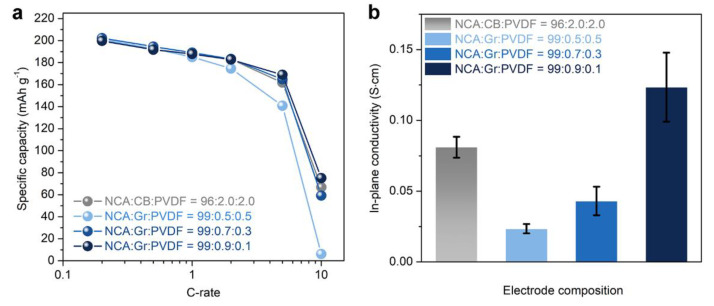
(**a**) Rate capability at 0.2–10 C and (**b**) in-plane conductivity of NCA electrodes with different electrode compositions. The ρ was set as ~3.8 g cm^−3^ and ~3.3 (±0.1) g cm^−3^ for Gr-containing electrodes and CB-containing electrodes, respectively.

We assessed rate capability of NCA electrodes that were fabricated using Gr/PVdF suspensions with different Gr contents (*x* in NCA:Gr:PVdF = 99:*x*:1−*x*) and compared a NCA electrode with a commercial-level electrode setting with NCA:CB:PVdF = 96:2:2 and *ρ*~3.3 g cm^−3^, which is hereafter denoted as the Ref. electrode. The discharge rate capability of the NCA electrodes measured at 0.2–10 C are shown in Figure 2a and the corresponding voltage profiles measured in the voltage range of 2.75–4.3 V vs. Li^+^/Li are shown in Figure S5. The NCA electrode containing 0.9 wt.% Gr exhibited good rate performance, comparable to that of the Ref. electrode: the two electrodes had ~200 and ~183 mAh g^-1^ at 0.2 C and 2 C, respectively. The NCA electrodes with less Gr contents (0.5 and 0.7 wt.%) showed slightly inferior performance, which could be attributed to the low electrical conductivities. These results were corroborated by in-plane conductivity and EIS measurements. As shown in Figure 2b, only the NCA electrode containing 0.9 wt.% Gr exhibited a comparable electrical conductivity with that of the Ref. electrode, whereas the others had low conductivities. In addition, we compared Nyquist plots of the NCA electrodes in configurations of a symmetric cell (NCA|NCA) and an asymmetric cell (Li|NCA), as depicted in Figure S6. The data for symmetric cells before the formation process (Figure S6a) revealed that the contact resistance between NCA particles and current collector and the interparticle resistance within electrodes was decreased with increasing Gr content [28,29]: the Nyquist plot of 0.9 wt.% Gr-NCA electrode was similar to that of the Ref. electrode. Similarly, for asymmetric cells after formation (Figure S6b), 0.9 wt.% Gr-NCA electrode and Ref. electrode had almost similar trends in the Nyquist plots, two depressed arcs at high frequencies and a straight line inclined at a constant angle to the real axis at low frequencies [30].

**Figure 3:**
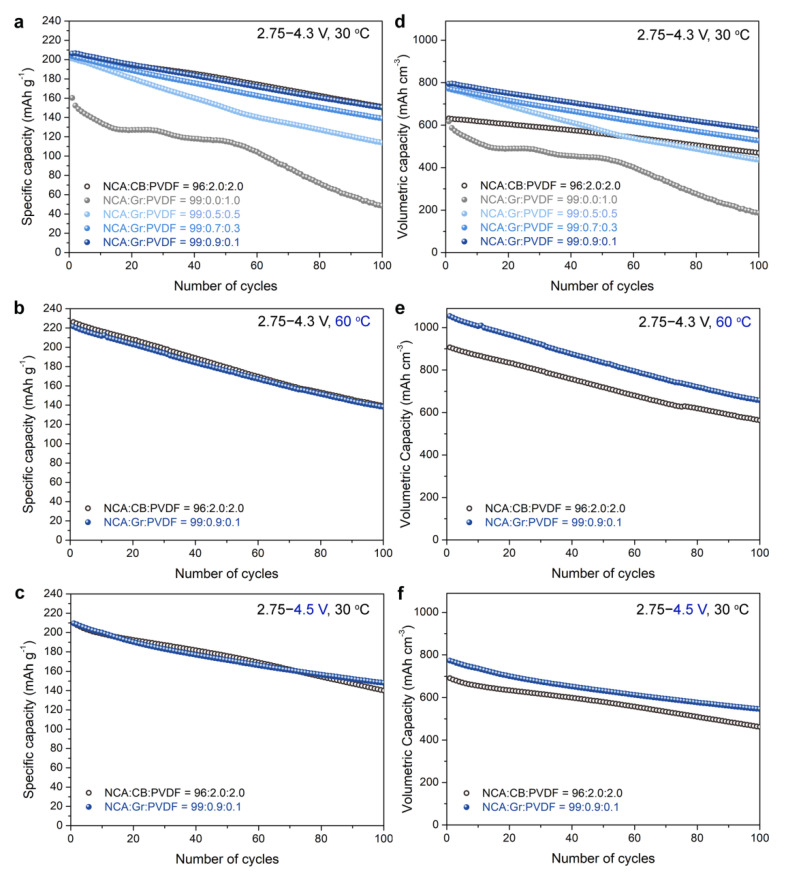
(**a**–**c**) Specific capacity retention and (**d**–**f**) volumetric capacity retention of NCA electrodes with different electrode compositions at current rate of 0.5 C, measured in the voltage range of (**a**,**d**) 2.75–4.3 V at 30 °C, (**b**,**e**) 2.75–4.3 V at 60 °C, and (**c**,**f**) 2.75–4.3 V at 30 °C.

Next, we evaluated the cycle performance of NCA electrodes with different electrode compositions, measured in the voltage ranges of 2.75–4.3 V or 2.75–4.5 V vs. Li^+^/Li at a current rate of 0.5C and temperatures of 30 °C and 60 °C. As shown in Figure 3a,b, the NCA electrode with 0.9 wt.% Gr exhibited a similar capacity retention to the Ref. electrode at 30 °C and 60 °C, respectively. With the decreasing Gr content, the capacity decay was severe. Under the operation at high-voltage cut-off condition (2.75–4.5 V vs. Li^+^/Li) and 30 °C, both the 0.9 wt.% Gr-NCA electrode and the Ref. electrode had similar behaviors during 100 cycles (Figure 3c). These results confirm that even with a high NCA content (99 wt.%), but low contents of inactive components (total 1 wt.%), the NCA electrodes show excellent cycling stability, comparable to the Ref. electrode containing high contents of CB/PVdF (total 4 wt.%). Note that the Gr-NCA electrodes had a considerably higher *ρ* (~3.8 g cm^−3^) than that of the Ref. electrode (*ρ*~3.3 g cm^−3^), featuring dense electrode architectures. By considering the electrode volume, we replotted the Q_vol_ retention data in Figure 3d–f. The NCA electrode with 0.9 wt.% Gr exhibited a considerably large Q_vol_, compared to that of the Ref. electrode. For example, at 30 ^°^C (Figure 3d), the 0.9 wt.% Gr-NCA electrode had a Q_vol_ of ~790 mAh cm^−3^ at the 1st cycle and delivered a Q_vol_ of ~580 mAh cm^−3^ at the 100th cycle (~73% retention), whereas the Ref. electrode had a Q_vol_ of ~610 mAh cm^−3^ and ~460 mAh cm^−3^ at 1st and 100th cycles, respectively, with capacity retention of ~75%.

**Figure 4:**
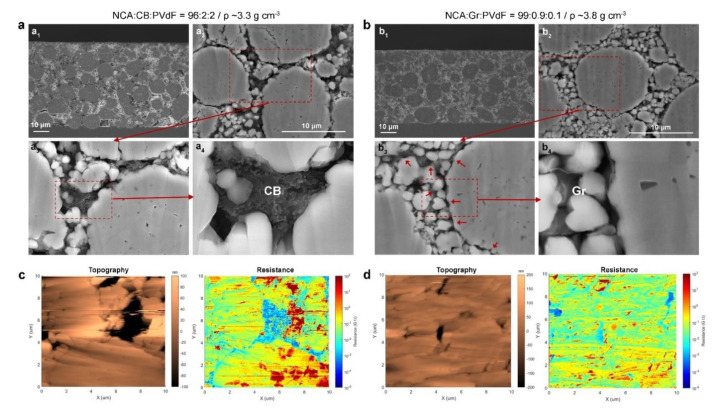
(**a_1_**−**a_4_**, **b_1_**−**b_4_**) Cross-sectional SEM images and (**c**,**d**) AFM and SSRM images of NCA electrodes with (**a**,**c**) CB/PVdF and (**b**,**d**) Gr/PVdF. The red arrows in b_3_ indicate Gr sheets between the NCA particles.

To differentiate the microstructure and components distribution for NCA electrodes with different compositions (1 wt.% Gr/PVdF vs. 4 wt.% CB/PVdF), we performed CP-SEM observation for 0.9 wt.% Gr-NCA and Ref. electrodes. As shown in Figure 4a_1_,b_1_, a distinct difference in the electrode thicknesses between the 0.9 wt.% Gr-NCA electrode and Ref. electrode was observed due to their different ρ. The Gr sheets were uniformly distributed within the Gr-NCA electrode, overall (Figure 4b_2_–b_4_). On the other hand, the Ref. electrode showed local agglomeration of CB nanoparticles between the NCA particles (Figure 4a_2_–a_4_). It is noteworthy that the 0.9 wt.% Gr-NCA electrode had no notable micro-cracks, even after a high-pressure calendaring process for high electrode density (3.8 g cm^−3^). We further conducted SSRM measurements combined with AFM on the cross section for the 0.9 wt.% Gr-NCA and Ref. electrodes, to visualize spatial heterogeneity of the resistance within NCA particles (see Figure 4c,d). Comparison between the resistance mapping images revealed that the Gr-NCA electrode had relatively uniform distributions of the intraparticle resistance, relative to those of the Ref. electrode. This result indicates that the use of Gr/PVdF suspension affords the uniform distribution of the conducting agent, Gr and hence, effective conducting pathways within NCA electrodes, even with a significantly small Gr amount (0.9 wt.%).

**Figure 5:**
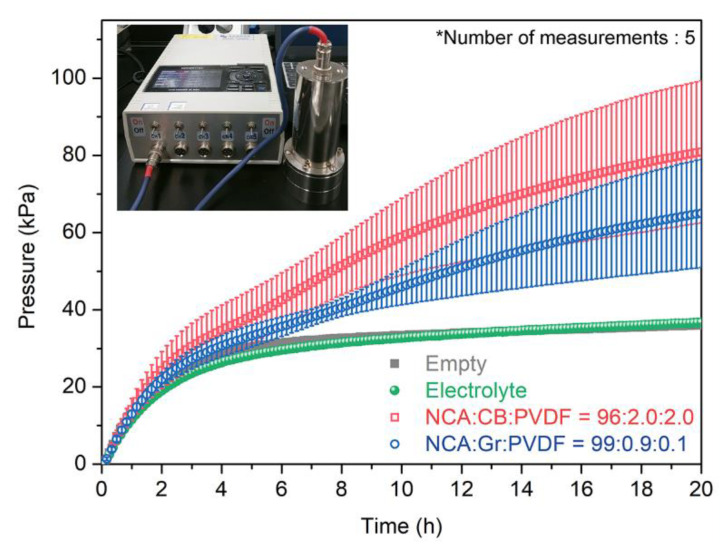
Internal pressure changes of in-situ gas cells, which contained NCA electrodes with CB/PVdF and Gr/PVdF (number of measurements: 5 times). Graphs for an empty cell and a cell containing only electrolyte as references are also shown.

Ni-rich oxide cathodes have been reported to cause safety issues due to structural instability arising from the phase transition during repeated cycles, accompanied by the evolution of O_2_ and CO_2_ gases [31,32]. This behavior is accelerated under battery operation with overcharging (>4.3 V vs. Li^+^/Li) and/or at elevated temperatures in the charged state. In addition, inactive materials such as CB and PVdF can release gaseous CO_2_ through oxidation reactions with reactive oxygen species generated from charged NCA particles [33]. Such gaseous byproducts could induce severe thermal runaway by highly exothermic reaction with flammable electrolytes. We monitored the internal pressure change with respect to testing time using a home-made gas cell system, which contained a NCA electrode fully charged to 4.5 V vs. Li^+^/Li and 10 mL of a fresh electrolyte, at 60 °C in Ar atmosphere (refer to the inset of Figure 5). An empty cell and a cell containing only electrolyte were also examined as references. As shown in Figure 5, as the testing proceeded, the internal pressure monotonically increased for all the cells. The NCA electrode containing Gr/PVdF exhibited a relatively small increase of the pressure, as compared to that of the Ref. electrode, which appeared to be a result of the use of less contents of inactive materials, confirming the efficacy of Gr/PVdF suspension.

**Figure 6:**
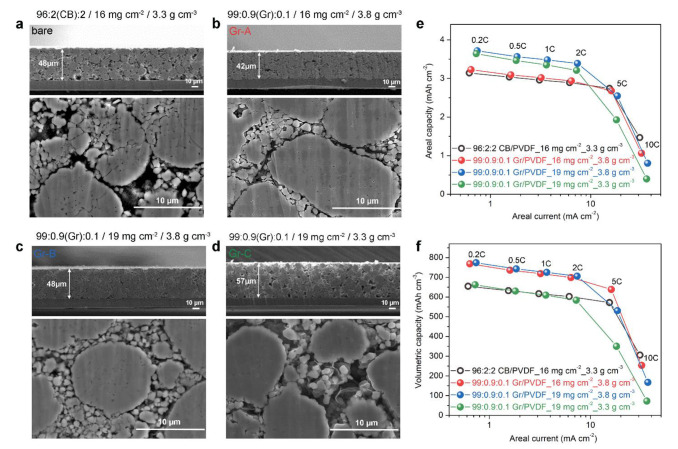
(**a**–**d**) Cross-sectional SEM images of CB- or Gr-containing NCA electrodes with different electrode parameters (Ref., Gr-A, Gr-B, and Gr-C) and (**e**,**f**) Q_areal_ and Q_vol_ with respect to areal current (at 0.2–10 C).

Lastly, we evaluated discharge Q_areal_ and Q_vol_ of NCA electrodes containing 1 wt.% Gr/PVdF. We fabricated three types of Gr-NCA electrodes (Gr-A, Gr-B, and Gr-C) with different electrode parameters, such as mass loading (m_areal_; 16 mg cm^−2^ vs. 19 mg cm^−2^) and electrode density, *ρ* (3.3 vs. 3.8 g cm^−3^), to compare with the Ref. electrode containing 4 wt.% CB/PVdF (m_areal_; 16 mg cm^−2^; *ρ* ~3.3 g cm^−3^). Figure 6a–d show the cross-sectional SEM images of the four electrodes with different electrode parameters. For the Ref. electrode (Figure 6a), the presence of intraparticle microcracks was prominent. On the other hand, overall, the microstructures of Gr-NCA electrodes with a high *ρ* of 3.8 g cm^−3^ (Gr-A and Gr-B; Figure 6b,c) and Gr-NCA electrode with the same *ρ* (3.3 g cm^−3^; Gr-C; Figure 6d) were intact after the calendaring process. These results confirm that the low-density CB-containing Ref. electrode with conventional electrode parameters have a limited *ρ* (≤3.3 g cm^−3^) and hence, a Q_vol_ limit [34,35]. The Q_areal_ and Q_vol_ of NCA electrodes with respect to areal current are depicted in Figure 6e,f, respectively. All of the electrodes showed monotonic capacity decreases with increasing areal current. The Gr-A electrode (red symbols) exhibited similar trend in the Q_areal_ decay with the Ref. electrode (black symbols). However, the Gr-A electrode delivered a considerably large Q_vol_ of ~768 mAh cm^−3^ (~17% increase) at 0.2C, compared to that of the Ref. electrode (Q_vol_ of ~655 mAh cm^−3^). It should be noted that for Gr-B electrode (blue symbols), both the Q_areal_ and Q_vol_ were larger than those of Ref. electrode at 0.2C–2C: the Q_areal_ and Q_vol_ of Gr-B were ~3.7 mAh cm^−2^ and ~774 mAh cm^−3^, respectively, at 0.2C. These results were ascribed to the high content of NCA (99 wt.%) while reducing inactive components (1 wt.% Gr/PVdF), which enabled for an enhanced *ρ* and hence, a large Q_vol_ (thinner electrode), as well as a large Q_areal_ (high mass-loading).

## 4. Conclusions

Electrochemically exfoliated Gr nanosheets were employed as conducting agent in NCA cathodes for LIBs. Gr/PVdF suspensions were fabricated by mixing Gr dispersion (in NMP) and PVdF solution (in NMP) at Gr contents of 10–90 wt.%. The Gr/PVdF suspensions appeared to be considerably homogeneous, compared to the CB/PVdF suspensions. Consequently, the cast Gr/PVdF composite films had uniform microstructures, which was thought to be due to specific interactions between the Gr sheets and PVdF, such as hydrogen bonding and π-π interactions. By using Gr/PVdF suspensions, we achieved highly packed NCA electrodes featuring a high NCA content of 99 wt.% and a high *ρ* of 3.8 g cm^−3^ without notable microcracks. The CP-SEM and AFM-SSRM analyses confirmed that the use of the Gr/PVdF suspension enabled the uniform distribution of Gr nanosheets, ensuring effective conducting pathways within NCA electrodes, even with an extremely small Gr amount (0.9 wt.%). In addition, the home-made gas cell measurements proved the efficacy of using a small content of Gr/PVdF (1 wt.%), as compared to the case of conventional CB/PVdF (4 wt.%), in terms of battery safety. The NCA electrodes using a small content of Gr/PVdF (0.9:0.1, total 1 wt.%) showed a larger Q_vol_ of ~790 mAh cm^−3^ than that of the Ref. electrode (Q_vol_~610 mAh cm^−3^) with a commercial-level of electrode setting (96 wt.% NCA; *ρ*~3.3 g cm^−3^) with good capacity retentions during 100 cycles, measured in the voltage ranges of 2.75–4.3 V and 2.75-4.5 V vs. Li^+^/Li and at temperatures of 30 °C at a current rate of 0.5 C. Furthermore, by enhancing the m_areal_ (19 mg cm^−2^) of the 0.9 wt.% Gr-containing NCA electrode, we demonstrated a high Q_areal_ of ~3.7 mAh cm^−2^ and a high Q_vol_ of ~774 mAh cm^−3^ at 0.2 C. These findings suggest that the use of Gr/PVdF suspensions is a promising solution for advanced Ni-rich oxide cathodes.

## Data Availability

The data that support the findings of this study are available from the corresponding author upon reasonable request.

## Supplementary Materials

The following are available online at https://www.mdpi.com/article/10.3390/ma14092271/s1, Figure S1: AFM data of Gr sheets, Figure S2: XPS C 1s of Gr sheets, Figure S3: Raman data of Gr sheets, Figure S4: Digital photographs of dispersions, Figure S5: Voltage profiles of NCA electrodes, Figure S6: Nyquist plots of NCA electrodes.
