# Supplementary material for: Graphene/PVDF Composites for Ni-rich Oxide Cathodes toward High-Energy Density Li-ion Batteries
Source: Materials (Basel). 2021 Apr 27;14(9):2271. doi: 10.3390/ma14092271 (PMC8124717; doi:10.3390/ma14092271)
Supplement: Supplementary file 1 [file materials-14-02271-s001.zip › materials-1176239-supplementary.pdf]

materials

MDPI

Supplementary Material

Graphene/PVDF Composites for Ni-rich Oxide Cathodes Toward High-Energy Density Li-ion Batteries

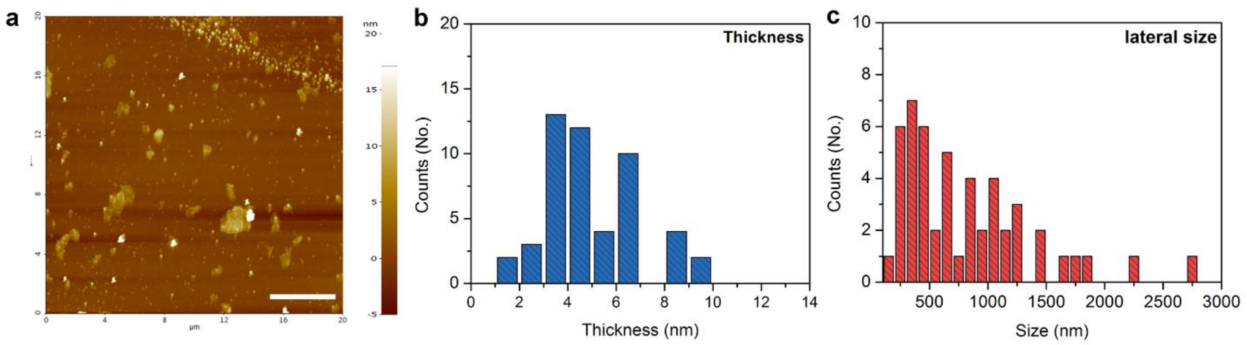

Figure S1. (**a**) AFM images with a scan area of 20 × 20 μm^2 and (**b**) thickness- and (**c**) lateral size-distributions of electrochemically exfoliated Gr sheets. Scale bar in **a**, 5 μm.

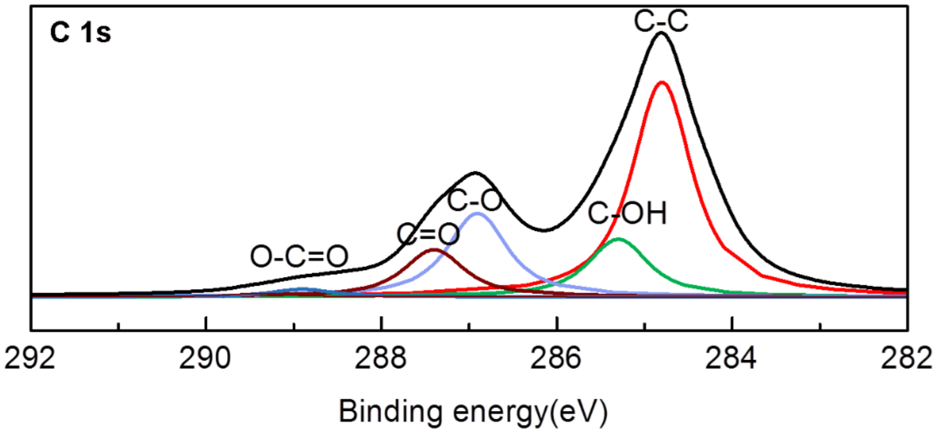

Figure S2. XPS C 1s spectra of as-exfoliated Gr nanosheets.

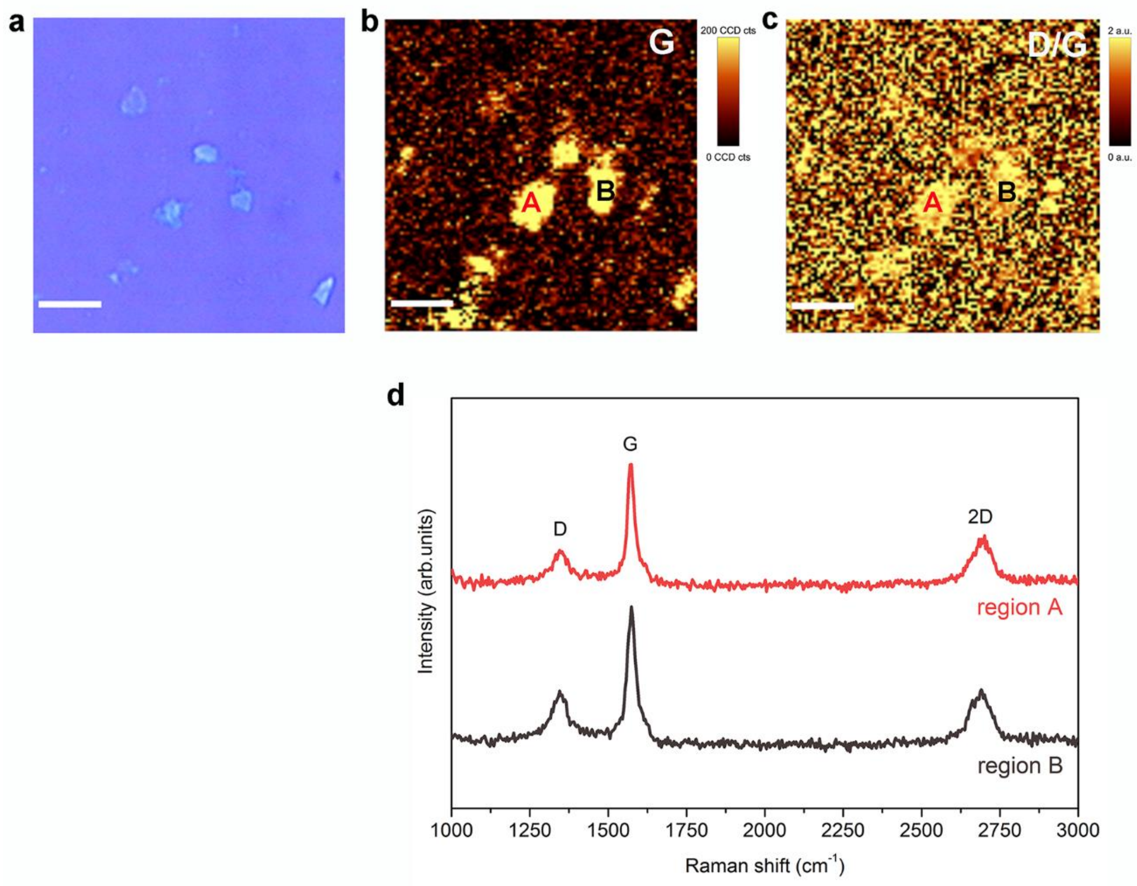

Figure S3. (**a**) Optical image; (**b,c**) Raman mapping images; and (**d**) Raman spectra of the corresponding regions on as-exfoliated Gr nanosheets. The Raman mapping images show contrasts of the G band (**b**) and D/G bands (**c**). All of the scale bars indicate 10 μm.

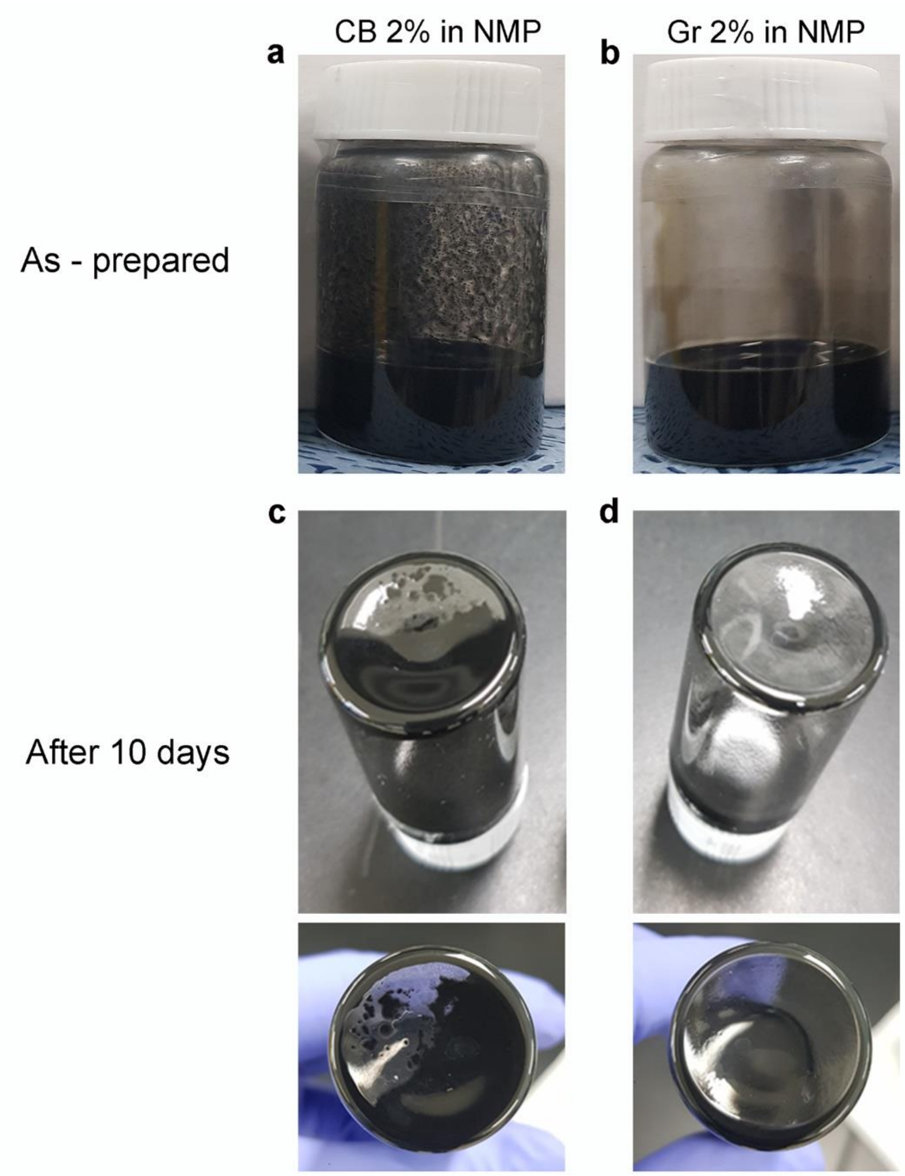

Figure S4. Digital photographs of (**a**,**c**) CB and (**b**,**d**) Gr dispersions (2 wt% in NMP) before and after 10 days.

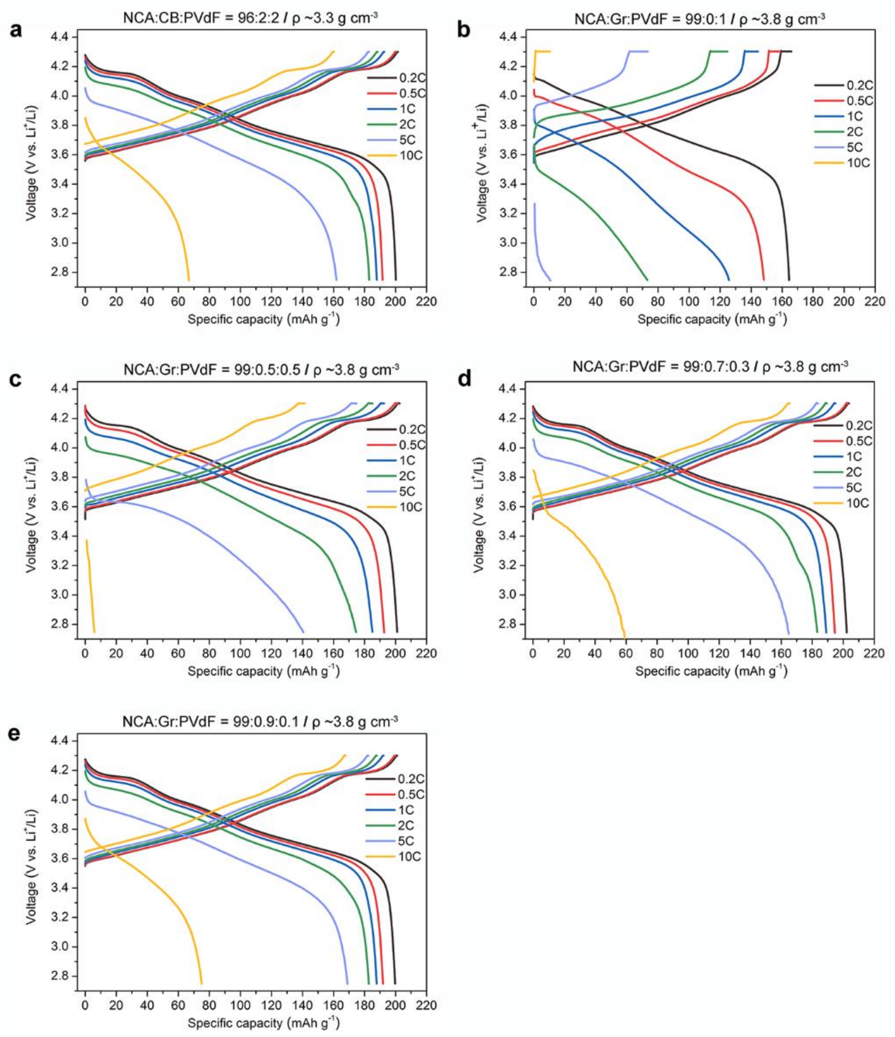

Figure S5. Voltage profiles of NCA electrodes with different electrode compositions and electrode densities (**a**–**e**), measured in the voltage range of 2.75–4.3 V vs. Li^+/Li at 0.2–10C and 30 °C.

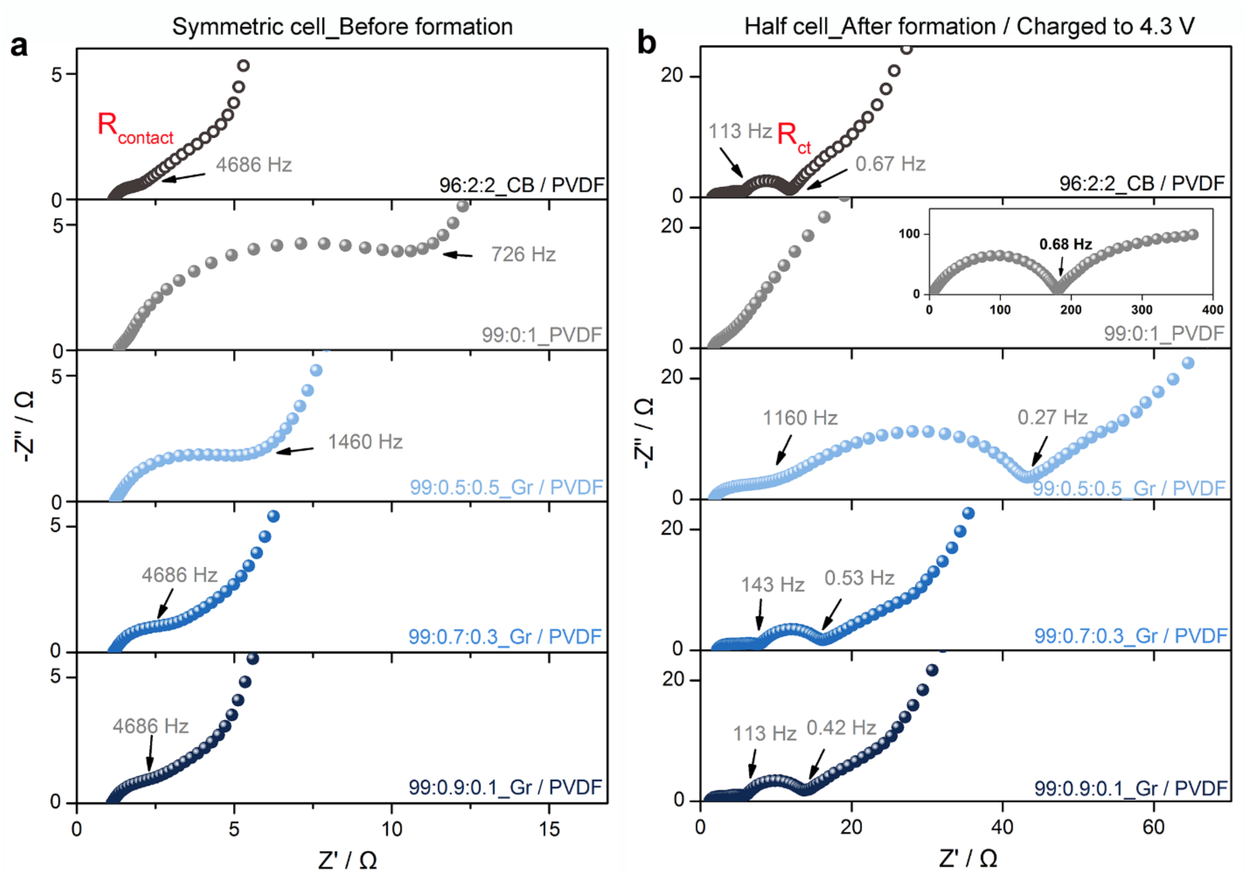

Figure S6. Nyquist plots of NCA electrodes with different electrode compositions, measured in configurations of (**a**) symmetric cells before formation and (**b**) asymmetric cells after formation and charged to 4.3 V vs. Li^+/Li. The arc in **a** indicate the contact resistance ($R_{contact}$) between NCA particles and current collector and the interparticle resistance within electrodes. The impedance spectra in **b** show depressed arcs at high frequencies and a straight line inclined at a constant angle to the real axis at low frequencies. The high- and medium-frequency arcs indicate the film resistance (R_f) and the charge-transfer resistance (R_{ct}), respectively.
